# Closed-loop temperature management with internet of things technology support in elderly laparoscopic rectal cancer surgery: A randomised controlled trial

**DOI:** 10.1371/journal.pone.0335993

**Published:** 2025-11-13

**Authors:** Xue Wang, Jiaqi Xu, Liqun Mo, Li Liu, Jun Zhou, Jun Jiang, Yingying Zhang, Yiping Bai

**Affiliations:** 1 Department of Operating Room, Panzhihua Central Hospital, Panzhihua, China; 2 Department of Operating Room, The Affiliated Hospital, Southwest Medical University, Luzhou, China; 3 Department of Anesthesiology, The Affiliated Hospital, Southwest Medical University, Luzhou, China; 4 Department of General Surgery (Thyroid Surgery), The Affiliated Hospital, Southwest Medical University, Luzhou, China; 5 Anesthesiology and Critical Care Medicine Key Laboratory of Luzhou, Luzhou, China; AdventHealth Orlando, UNITED STATES OF AMERICA

## Abstract

**Background:**

This study investigates the effectiveness of closed-loop temperature management supported by Internet of Things technology in elderly patients undergoing laparoscopic rectal cancer surgery.

**Methods:**

Eighty-nine patients were randomly assigned to three Groups. The Enhanced with Warming Blanket Group utilized inflatable warming blankets for insulation. The Closed-Loop Thermoregulation Group employed real-time core temperature monitoring and temperature regulation supported by IoT technology. Core temperatures were continuously monitored from entering the operating room to departure from the Post-Anesthesia Care Unit. Postoperative outcomes including time to tracheal extubation, length of stay in the PACU, incidence of postoperative shivering, time to first flatus, time to first oral feeding, and postoperative length of hospital stay were recorded and compared.

**Results:**

From 30 minutes after anesthesia induction to departure from the PACU, the Closed-Loop Thermoregulation Group exhibited significantly higher core body temperature than the Routine Group (*All P* < 0.05). The incidence of hypothermia upon entering and departure from the PACU was significantly lower in the Closed-Loop Thermoregulation Group compared to the Routine Group (*P* = 0.005; *P* = 0.005). The proportion of time spent in hypothermia was significantly lower in both the Enhanced with Warming Blanket Group and the Closed-Loop Thermoregulation Group compared to the Routine Group (*P* < 0.001; *P* < 0.001). Compared to the Routine Group, both the Enhanced with Warming Blanket Group and the Closed-Loop Thermoregulation Group had significantly shorter times to tracheal extubation (P = 0.033; *P* = 0.006) and length of stay in the PACU (*P* < 0.001; *P* < 0.001), as well as significantly reduced incidence of postoperative shivering (*P* = 0.010; *P* = 0.012).

**Conclusions:**

IoT-supported closed-loop temperature management maintains core temperatures, reduces hypothermia and shivering, and shortens extubation and recovery times in elderly laparoscopic rectal cancer patients effectively.

## Introduction

Laparoscopic radical rectal cancer surgery is the preferred clinical treatment option for patients with rectal cancer [1]. However, advanced age, extensive surgical trauma, prolonged duration, and the disruptive effects of anesthetic agents on temperature regulation significantly increase the risk of intraoperative and postoperative hypothermia [2,3]. Hypothermia can lead to a range of complications, including cardiovascular events, increased intraoperative bleeding, postoperative shivering, delayed emergence, and ultimately, adverse patient outcomes [4–7]. Although various warming measures have been employed clinically, enhanced integration of temperature monitoring and regulation remains imperative. Conventional devices such as inflatable warming blankets exhibit rigid temperature control functionality and lack granular adjustment capabilities, thereby raising safety concerns identified by scholars. Specifically, excessively high outlet temperatures or uneven airflow distribution may induce localized thermal tissue injury through prolonged exposure. Furthermore, their dependence on fixed temperature thresholds within an open-loop control architecture—devoid of real-time feedback—increases risks of overheating or incomplete rewarming [8,9]. To directly address these specific safety concerns, this study introduces IoT to construct a closed-loop management system comprising wireless core body temperature monitoring, data management, and self-regulating warming intervention. This system enables: 1) Continuous and accurate core temperature monitoring, which provides reliable real-time data on the patient’s thermal status; 2) Dynamic adjustment of the warming device’s output power and airflow parameters (temperature/distribution) based on real-time temperature data, enabling precise and personalized regulation to mitigate the risk of localized overheating caused by fixed high settings or uneven airflow; 3) Establishing a “ monitoring-analysis-intervention-remonitoring ” closed loop, ensuring body temperature is consistently maintained within the target range, effectively preventing both systemic overheating and incomplete rewarming. We aim to validate the clinical application effectiveness of this system through our study, providing evidence to support the reduction of perioperative hypothermia and associated complications, advancing ongoing improvements in operating room temperature management, and ultimately improving patient outcomes.

## Materials and methods

### 1. Trial design and registration

Approval was obtained from the Institutional Review Board of The Affiliated Hospital, Southwest Medical University (KY2020078; May 29, 2020). The trial was registered at www.chictr.org.cn on June 11, 2020 (ChiCTR2000033763; principal investigator: Xue Wang, M.D.). This prospective randomized single-blind study was conducted from June to October 2020.

### 2. Participants

**Fig 1:**
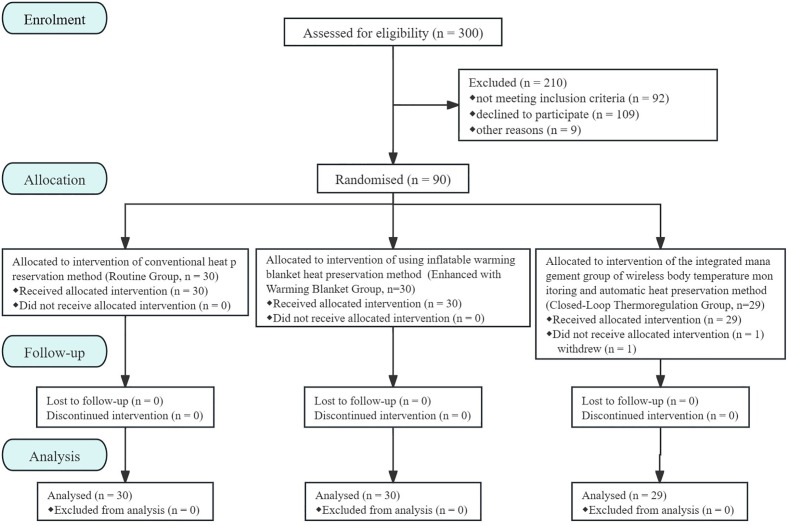
Subjects enrollment and randomization. Patients in Routine Group receive conventional warming methods, patients in Enhanced with Warming Blanket Group receive conventional warming along with the use of an inflatable warming blanket, and patients in Closed-Loop Thermoregulation Group receive conventional warming along with wireless core body temperature monitoring and integrated self-regulated warming management.

A total of 89 patients aged ≥18 years, ASA I-II, with a preoperative morning body temperature <37.5°C and ≥36.0°C, scheduled for elective laparoscopic radical resection of rectal cancer, were included (Fig 1). Exclusion criteria were as follows: 1) fever within 3 days before surgery; 2) temperature regulation abnormalities such as malignant hyperthermia, neuroleptic malignant syndrome; 3) hyperthyroidism; 4) conversion to open surgery; 5) emergency surgery; 6) patient refusal or non-cooperation.

### 3. Interventions

**Fig 2:**
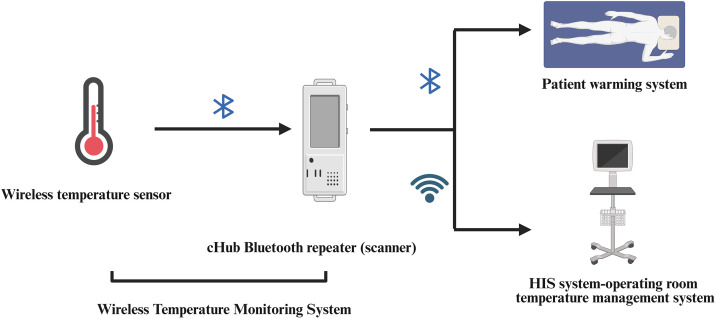
Schematic Diagram of Core Body Temperature Closed-Loop Management. We employ a closed-loop temperature management approach during surgery utilizing wireless monitoring, data transmission, and automatic heat preservation intervention. Created in BioRender. Jiaqi, **X.** (2025) https://BioRender.com/1rucg66.

The Routine Group received conventional thermal measures, with room temperature maintained at 22–24°C, humidity controlled at 40–60%, the body outside the surgical area covered with blankets, and the intraoperative irrigation fluid heated to 38°C. In addition to routine measures, the Enhanced with Warming Blanket Group utilized a Forced-Air Warming device (FAW, Bair Hugger model 750, Arizant Healthcare) for warming, with FAW temperature set to the medium temperature (38°C ± 1.5°C) intraoperatively. The blankets in the latter two groups were applied to both the upper body and lower body. The closed-loop thermoregulation group adopted a combination of conventional heat preservation and the “integrated closed-loop mode of core body temperature monitoring and self-controlled heat preservation” for heat preservation and established a body temperature management mode: With the aid of Wi-Fi, Bluetooth, QR codes and information technology, the mutually independent wireless body temperature monitoring system, patient warming system and operating room information management system were connected and interconnected to form an information closed loop, that is, to implement a three-dimensional linkage of “core body temperature monitoring - data transmission and analysis - heat preservation intervention” Personalized and self-controlled heat preservation closed-loop management. A wireless temperature sensor was placed in the axilla, which has a good correlation with the core body temperature of the esophagus [10]. Before the patient entered the operating room, the carbon fiber blanket in the patient warming system on the operating table was preheated for 15 minutes to reach the preset heat preservation value of 38°C. At the same time, the core body temperature target value for heat preservation and maintenance during the operation of the patient was preset on the warming system to be 36°C to 37°C. The patient warming system (Warm6200, Beijing Yingtai Nuo Medical Technology Co., Ltd.) automatically adjusted the heating power parameters based on the data of the wireless temperature sensor (read once per minute) and the preset core body temperature target, thereby achieving real-time management of heat preservation and temperature maintenance (Fig 2).

### 4. Outcomes

Core body temperature and the occurrence of hypothermia as the primary outcome measures. Core body temperature of patients was continuously monitored from entering the operating room to departure from the PACU. Core body temperature was compared at various time points: upon entering the operating room, at anesthesia induction, every 15 minutes post-anesthesia induction up to 180 minutes, at the end of surgery, upon admission to the PACU, and upon departure from the PACU. Time to tracheal extubation, length of stay in the PACU, incidence of postoperative shivering, time to first flatus, time to first oral feeding, and postoperative length of hospital stay were recorded and compared.

### 5. Randomization and blinding

Randomization: computer-generated sequence (block size 6) by independent statistician. Allocation concealed via sequentially numbered, opaque, sealed envelopes. Envelopes opened by neutral staff after anesthesia induction**. Blinding:** patients and outcome assessors blinded to group allocation. Perioperative care teams unavoidably unblinded due to device visibility.

### 6. Statistical analysis

The study employed a parallel-group design with the core body temperature as the primary outcome measure. The sample size per group was calculated using the formula: n = λ/Δ, where $\Delta =\frac{1}{\sigma^{2}}\sum_{i=1}^{k}{\left(u_{i}-u_{0}\right)}^{2}$. The symbols are defined as follows: n denotes the sample size per group; λ represents a constant derived from statistical tables based on α, β, and the number of groups (k); Δ is the effect size parameter; σ is the pooled standard deviation; k is the number of groups; u_i_ is the mean core body temperature of group i and u₀ is the overall mean core body temperature across all groups. Pilot data showed that the mean core body temperatures were 35.84°C for the routine group (u1), 36.16°C for the Enhanced with Warming Blanket Group (u2), and 36.55°C for the closed-loop thermoregulation group (u3), with a pooled standard deviation (σ) of 0.65°C. The overall mean (u₀) was 36.18°C, and the effect size (Δ) was calculated as 0.60. For α = 0.05, β = 0.1, and k = 3, the constant λ from the table was 12.66, leading to an initial sample size per group of n = 12.66/0.60 = 21.1, which was rounded up to 22. After accounting for a 20% dropout rate, the final sample size per group was adjusted to 30 patients.

Statistical analysis was performed using IBM SPSS Statistics version 25.0. Descriptive statistics are reported as mean ± standard deviation, number (percentage), or median [interquartile range]. Parametric data, meeting assumptions of normality and homogeneity of variances, underwent analysis of variance (ANOVA) for multiple group comparisons, while non-parametric tests were utilized for data not meeting these assumptions. Specifically: for continuous variables (non-normally distributed) or ordinal variables: Kruskal-Wallis test was applied; for categorical variables (when sample size was small or expected cell frequency <5): Fisher’s exact test was used. Comparisons of core body temperature among the three patient groups at individual time points were analyzed using one-way ANOVA, while comparisons across multiple time points were analyzed using repeated-measures ANOVA. Count data were analyzed using chi-square test or Fisher’s exact test. Significance level was set at a two-sided P value less than 0.05, indicating statistical significance.

## Results

A total of 90 patients were enrolled in this study, with 89 patients ultimately included in the analysis**.** There were no statistically significant differences in baseline characteristics among the three groups (Table 1).

**Table 1:** Demographics and characteristics of the three groups of patients.

Characteristics	Routine group (n = 30)	Enhanced with warming blanket group (n = 30)	Closed-loop thermoregulation group (n = 29)	*P*
**Age (years), mean ± SD**	60.87 ± 10.19	62.03 ± 13.77	62.79 ± 9.75	0.870
**Sex (male/female)**	19/11	18/12	19/10	0.907
**ASA physical status, n (%)**				0.238
**Ⅰ**	27 (90.0)	27 (90.0)	29 (100.0)	
**Ⅱ**	3 (10.0)	3 (10.0)	0 (0.0)	
**BMI (kg/m²), mean ± SD**	22.51 ± 3.55	22.67 ± 4.14	24.94 ± 5.67	0.096
**Surgical method (Dixon/ Miles)**	25/5	25/5	22/7	0.702
**Comorbidities, n (%)**				
**Diabetes**	1 (3.3)	5 (16.7)	1 (3.4)	0.206
**Hypertension**	12 (40.0)	9 (30.0)	9 (31.0)	0.667

Values are expressed as mean ± SD or absolute number n (%). Continuous variables (Age, BMI) meeting normality assumptions were compared using one-way ANOVA; categorical variables (Sex, ASA status, Surgical method, Comorbidities) were analyzed by chi-square test or Fisher’s exact test.

**Fig 3:**
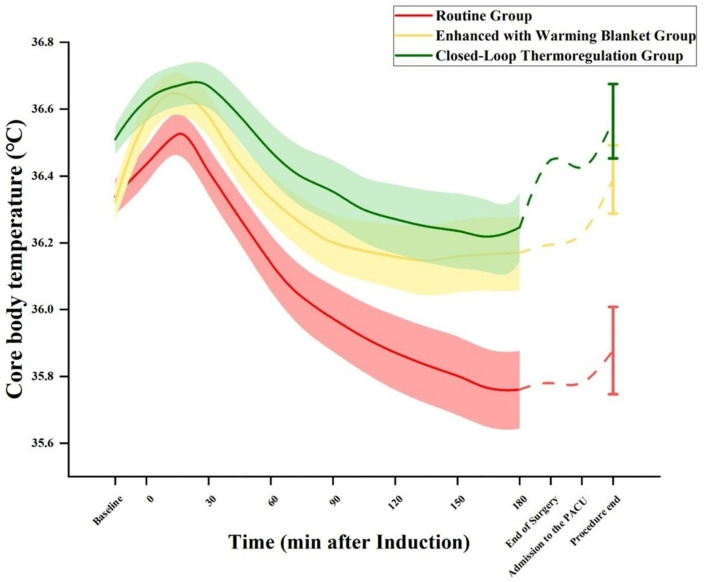
Core body temperature shown as a function of time following anaesthesia induction. Solid lines represent the mean across Routine (red, n = 30) and Enhanced with Warming Blanket (yellow, n = 30) and Closed-Loop Thermoregulation (green, n = 29) groups and shaded regions are SEM. Each patient’s baseline core body temperature was the average core body temperature one minute prior to induction.

The main result of this study concerns core body temperature. Significant differences in core body temperature were observed at different time points among the three groups (*P* = 0.002). From 30 minutes after anesthesia induction to departure from the PACU, the Closed-Loop Thermoregulation Group exhibited significantly higher core body temperature than the Routine Group (*All P* < 0.05); from 165 minutes after anesthesia induction to departure from the PACU, the Enhanced with Warming Blanket Group showed significantly higher core body temperature than the Routine Group (*All P* < 0.05). Throughout the observation period, there was no statistically significant difference between the Enhanced with Warming Blanket Group and the Closed-Loop Thermoregulation Group (*All P* > 0.05) (Fig 3 and Table 2).

**Table 2:** Comparison of core body temperature of three groups of patients at different time points.

Time points	Routine group (n = 30)	Enhanced with warming blanket group (n = 30)	Closed-loop thermoregulation group (n = 29)
**Entering the Operating Room**	36.38 ± 0.24	36.34 ± 0.25	36.48 ± 0.22
**Anesthesia induction**	36.43 ± 0.30	36.56 ± 0.35	36.62 ± 0.33
**15min after Anesthesia induction**	36.52 ± 0.33	36.63 ± 0.36	36.66 ± 0.33
**30min after Anesthesia induction**	36.41 ± 0.39	36.58 ± 0.34	36.66 ± 0.35^#^
**45min after Anesthesia induction**	36.27 ± 0.42	36.43 ± 0.35	36.58 ± 0.41^#^
**60min after Anesthesia induction**	36.13 ± 0.46	36.33 ± 0.38	36.47 ± 0.45^#^
**75min after Anesthesia induction**	36.04 ± 0.50	36.26 ± 0.42	36.39 ± 0.49^#^
**90min after Anesthesia induction**	35.97 ± 0.54	36.20 ± 0.45	36.35 ± 0.51^#^
**105min after Anesthesia induction**	35.91 ± 0.57	36.17 ± 0.48	36.30 ± 0.52^#^
**120min after Anesthesia induction**	35.87 ± 0.61	36.15 ± 0.52	36.27 ± 0.56^#^
**135min after Anesthesia induction**	35.83 ± 0.63	36.14 ± 0.56	36.25 ± 0.58^#^
**150min after Anesthesia induction**	35.80 ± 0.64	36.15 ± 0.59	36.06 ± 0.63^#^
**165min after Anesthesia induction**	35.76 ± 0.64	36.16 ± 0.59^*^	36.21 ± 0.58^#^
**180min after Anesthesia induction**	35.76 ± 0.64	36.17 ± 0.61^*^	36.25 ± 0.55^#^
**End of Surgery**	35.77 ± 0.72	36.20 ± 0.64^*^	36.45 ± 0.59^#^
**Admission to the PACU**	35.78 ± 0.76	36.22 ± 0.65^*^	36.42 ± 0.60^#^
**Departure from the PACU**	35.78 ± 0.71	36.39 ± 0.56^*^	36.56 ± 0.60^#^
**Overall P value (among groups): 0.002**			

Values are expressed as the Mean ± SD. Comparisons of core body temperature among the three patient groups at individual time points were analyzed using one-way ANOVA, while comparisons across multiple time points were analyzed using repeated-measures ANOVA. Symbols indicate significant difference compared to the Routine Group: * Enhanced with Warming Blanket Group, # Closed-Loop Thermoregulation Group (*All P* < 0.05).

Upon admission to and departure from the PACU, the incidence of hypothermia in the Closed-Loop Thermoregulation Group was significantly lower than that in the Routine Group (*P* = 0.005, *P* = 0.005), and the incidence of hypothermia in the Enhanced with Warming Blanket Group upon departure from the PACU was also significantly lower than that in the Routine Group (*P* = 0.015). The proportion of time spent in hypothermia was significantly lower in the Enhanced with Warming Blanket Group and the Closed-Loop Thermoregulation Group compared to the Routine Group (*P* < 0.001, *P* < 0.001); there was no statistically significant difference between the Enhanced with Warming Blanket Group and the Closed-Loop Thermoregulation Group (*P* = 0.851). There was no statistically significant difference in the severity of hypothermia among the three groups (*P* = 0.064, **Table 3**).

**Table 3:** Comparison of the occurrence of hypothermia among the three groups of patients.

	Routine group (n = 30)	Enhanced with warming blanket group (n = 30)	Closed-loop thermoregulation group (n = 29)	*P*
**Incidence of intraoperative hypothermia (n, %)**	20(66.7%)	15 (50.0%)	14 (48.3%)	0.289
**Incidence of hypothermia upon admission to the PACU (n, %)**	17 (56.7%)	12 (40.0%)	6 (20.7%) *	0.018
**Incidence of hypothermia at the time of leaving the PACU (n, %)**	15 (50.0%)	6 (20.0%) *	4 (13.8%) *	0.007
**Average duration of temperature monitoring (min,** $\overset{―}{x}\pm s$)	306.8 ± 61.6	290.8 ± 38.9	310 ± 51.27	<0.001
**Average duration of hypothermia (min,** $\overset{―}{x}\pm s$)	213.5 ± 81.9	61.1 ± 32.4	69.3 ± 41.2	
**Proportion of time spent in hypothermia (**$\overset{―}{x}\pm s$)	0.69 ± 0.20	0.21 ± 0.11*	0.22 ± 0.11*	
**Mild hypothermia (n, %)**	12 (60.0%)	14 (93.3%)	13 (92.9%)	0.064
**Moderate hypothermia (n, %)**	7 (35.0%)	1 (6.7%)	1 (7.1%)	
**Severe hypothermia (n, %)**	1 (5.0%)	0 (0)	0 (0)	

Values are expressed as mean ± SD or absolute number (%). Incidence of hypothermia (categorical) was analyzed by chi-square or Fisher’s exact test; the proportion of time spent in hypothermia (continuous) used one-way ANOVA; severity of hypothermia (ordinal) was compared using Kruskal-Wallis test. Compared to **Routine Group**, * *P* < 0.05. **Hypothermia** is characterized by a core body temperature dipping below 36°C for various reasons. Depending on the severity, hypothermia is categorized as mild (35.0 to 35.9°C), moderate (34.0 to 34.9°C), and severe (≤33.9°C). **The proportion of time spent in hypothermia** is defined as the total duration of hypothermia occurrences from the time the patient enters the operating room to the time they depart from the PACU, divided by the total duration of temperature monitoring.

Compared to the Routine Group, both the Enhanced with Warming Blanket Group and the Closed-Loop Thermoregulation Group exhibited significantly shortened time to tracheal extubation (*P* = 0.033; *P* = 0.006) and length of stay in the PACU (*P* < 0.001; *P* < 0.001), as well as significantly reduced incidence of postoperative shivering (*P* = 0.010, *P* = 0.012, **Table 4**).

**Table 4:** Comparison of the postoperative recovery among the three groups of patients.

	Routine Group (n = 30)	Enhanced with Warming Blanket Group (n = 30)	Closed-Loop Thermoregulation Group (n = 29)	*P*
**Time to tracheal extubation (min)**	12.07 ± 4.73	8.70 ± 6.83*	7.86 ± 2.46*	0.004
**Length of stay in the PACU (min)**	34.57 ± 6.11	28.06 ± 4.75*	28.43 ± 5.38*	<0.001
**Incidence of postoperative shivering (n, %)**	10 (33.3%)	2 (6.7%) *	2 (6.9%) *	0.011
**Time to first flatus [day/ M (P25, P75)]**	3.0 (2.8,3.0)	3.0 (3.0,5.0)	3.0 (2.5,4.0)	0.309
**Time to first oral feeding [day/ M (P25, P75)]**	4.5 (3.0,5.0)	4.5 (3.0,5.3)	4.0 (3.0,4.5)	0.104
**Postoperative length of hospital stay. [day/ M (P25, P75)]**	8.0 (7.0,9.3)	9.0 (8.0,11.0)	8.0 (7.0,10.0)	0.189

Values are expressed as mean ± SD, absolute number (%) or median [IQR]. Time to extubation and PACU stay (normally distributed) were compared by one-way ANOVA; incidence of shivering (categorical) used Fisher’s exact test; time to flatus, oral feeding, and hospital stay (non-normally distributed) were analyzed using Kruskal-Wallis test. Compared to Routine Group, * *P* < 0.05. **Time to tracheal extubation** is defined as the time interval between the completion of surgery and the removal of the endotracheal tube. **The length of stay in the PACU** is defined as the time interval between admission to and departure from the PACU. **The incidence of postoperative shivering** refers to the frequency or occurrence rate of involuntary body shivering or chills that occurs after surgery. **Time to first flatus** is defined as the time interval between the end of surgery and the occurrence of the first passage of flatus. **Time to first oral feeding** is defined as the time interval from the end of surgery to the initiation of the first oral intake of food or liquid. **Postoperative length of hospital stay** is defined as the time interval from the immediate completion of surgery to the patient’s formal discharge from the hospital.

There were no significant differences between the Enhanced with Warming Blanket Group and the Closed-Loop Thermoregulation Group in time to tracheal extubation, length of stay in the PACU, and incidence of shivering postoperatively (*All P* = 1.000). There were no statistically significant differences in time to first flatus, time to first oral feeding, and length of hospital stay among the three groups (*P* = 0.309, *P* = 0.104, *P* = 0.189, **Table 4**).

## Discussion

Under the application of IoT-supported closed-loop temperature management in elderly patients undergoing laparoscopic rectal cancer surgery, compared with the Routine Group, 1) it effectively maintains stable intraoperative core body temperature; 2) reduces the incidence of perioperative hypothermia and postoperative shivering; and 3) shortens the time to tracheal extubation and anesthesia recovery.

Core body temperature is crucial for assessing the condition of surgical patients [11]. Factors such as anesthesia and exposure during surgery predispose patients to hypothermia, impacting recovery [12]. This study found distinct trends in core body temperature decline among the groups. Thirty minutes after anesthesia induction, the core temperature of patients in the Routine Group was significantly lower than in the Closed-Loop Thermoregulation Group, suggesting better early efficacy of the closed-loop system. By 165 minutes, the Routine Group temperature was also significantly lower than the Enhanced with Warming Blanket Group, emphasizing the importance of active warming in prolonged surgery.

In this study, despite employing different warming strategies for the three groups of patients, the incidence of intraoperative hypothermia remained high. The incidence rates of intraoperative hypothermia were 66.7%, 50%, and 48.3% for the Routine Group, Enhanced with Warming Blanket Group, and Closed-Loop Thermoregulation Group, respectively. The high incidence of intraoperative hypothermia is consistent with previous studies by Santos et al, indicating it as a prevalent issue [13]. This higher incidence of hypothermia is likely closely associated with prolonged general anesthesia and the inherent complexity of laparoscopic rectal cancer surgery. General anesthesia suppresses patients’ thermoregulatory mechanisms, making them more susceptible to temperature fluctuations from the external environment. Additionally, the complexity of laparoscopic rectal cancer surgery itself may lead to longer surgical times; all three groups of patients in this study had surgeries lasting over 3 hours, thereby increasing the risk of temperature decline. This underscores the necessity of effective warming measures during prolonged surgical procedures.

We observed differences in the incidence of hypothermia, with the Closed-Loop Thermoregulation Group exhibiting lower rates both upon admission to and departure from the PACU compared to the Routine Group, while the Enhanced with Warming Blanket Group only showed lower rates upon departure from the PACU. This indicates a significant effect of closed-loop management in maintaining stable patient temperature. Further analysis revealed that the proportion of time spent in hypothermia was significantly higher in the Routine Group compared to the other two groups, highlighting the inadequacy of traditional warming methods in temperature maintenance. Although there was no significant difference between the Enhanced with Warming Blanket Group and the Closed-Loop Thermoregulation Group in the proportion of time spent in hypothermia, the closed-loop system could respond rapidly and precisely adjust based on real-time temperature changes, ensuring temperature remained within the normal range and minimizing the duration of hypothermia. This dynamic adjustment is difficult to achieve with the Enhanced with Warming Blanket Group, as it can only provide static, singular warming measures without the ability to adjust based on real-time changes in patient temperature [14].

Analysis of hypothermia severity revealed limited efficacy of routine measures (mild: 60%, moderate: 35%, severe: 5%). The Enhanced with Warming Blanket Group and the Closed-Loop Thermoregulation Group demonstrated better warming effects, with the incidence of moderate hypothermia reduced to 6.7% and 7.1%, respectively. Prior research has shown that even mild hypothermia can increase blood loss and the risk of postoperative infection [15–17], and both active warming methods effectively reduced moderate/severe hypothermia. Time to tracheal extubation and PACU stay were longer in the Routine Group. Early extubation reduces complications [18–20]. Both active warming methods reduced postoperative shivering. However, no significant differences were found in time to first flatus, oral feeding, or hospital stay, indicating need for further research on interdisciplinary recovery strategies.

## Study limitations

This study has certain limitations. The relatively small sample size and the exclusive focus on patients undergoing rectal cancer radical surgery restrict the generalizability. The absence of long-term follow-up data hinders a thorough assessment of the long-term effects of the warming strategies. The superiority of the Closed-Loop Thermoregulation Group over the Enhanced with Warming Group wasn’t fully clarified. Sensor errors pose risks, as relying on single-point axillary temperature measurements can be inaccurate due to environmental and positional factors. Technical issues and the need for regular support, calibration, and operator training are significant. There’s also a potential overheating risk, especially for specific patient groups.

## Conclusion

This randomized trial demonstrates that IoT-supported closed-loop temperature management effectively maintains core body temperature, reduces perioperative hypothermia and shivering, and accelerates recovery in elderly patients undergoing laparoscopic rectal cancer surgery. Compared to conventional warming, this approach provides earlier and more precise thermal stabilization. These findings support the integration of closed-loop systems into standard perioperative care for high-risk populations to improve clinical outcomes. Future research should focus on optimizing the technology for broader surgical applications.

## Supporting information

S1 File CONSORT checklist. (DOC)

S2 File Research proposal (English). (DOCX)

S3 File Research proposal (Chinese). (DOC)

S4 File Study data. (XLSX)

S5 File Closed-loop thermoregulation group. (XLSX)

S6 File Enhanced with warming blanket group. (XLSX)

S7 File Routine group. (XLSX)
