# Supplementary material for: Closed-loop temperature management with internet of things technology support in elderly laparoscopic rectal cancer surgery: A randomised controlled trial
Source: PLoS One. 2025 Nov 13;20(11):e0335993. doi: 10.1371/journal.pone.0335993 (PMC12614596; doi:10.1371/journal.pone.0335993)
Supplement: S2 File — (DOCX) [file pone.0335993.s002.docx]

**Research Proposal**

**Project Name (Chinese):** 无线体温监测与智能保温一体化管理模式在腹腔镜直肠癌根治术中的应用研究

**Project Name (English):** Application of Wireless Temperature Monitoring and Intelligent Insulation Management in Laparoscopic Radical Resection of Rectal Cancer

**Research Institution:** The Affiliated Hospital, Southwest Medical University, Luzhou, China

**Principal Investigator:** Xue Wang

**Sponsor:** The Affiliated Hospital, Southwest Medical University, Luzhou, China

**I. Research Background**

Body temperature is one of the vital signs essential for maintaining normal metabolism and physiological functions. Abnormal body temperature can lead to metabolic disorders and even life-threatening conditions. Surgical patients are particularly prone to intraoperative or postoperative hypothermia due to factors such as the specific environment of the operating room, exposure of surgical sites, surgical procedures, anesthesia, infusion of large volumes of room-temperature or cold fluids, and irrigation of the surgical field. However, limitations in conventional temperature monitoring methods and insufficient availability of warming devices often result in low compliance with intraoperative temperature monitoring and inadequate or excessive warming, which can significantly impact postoperative recovery.

Recently, the integration of internet and AI technologies has enabled the development of an intelligent temperature monitoring and warming management system. This system combines patient warming devices with wireless temperature monitoring sensors, forming a closed-loop management system for temperature monitoring and warming. Specifically, wireless temperature sensors transmit data via Bluetooth converters to the Warm6200 controller, which manages patient temperature based on real-time data. This innovative approach is expected to revolutionize clinical temperature management in terms of concepts, methods, and outcomes. However, its clinical efficacy and optimal utilization remain to be explored, especially in the context of laparoscopic radical resection of rectal cancer.

Our research team's previous cross-sectional survey revealed an intraoperative hypothermia incidence rate of 66.3% in colorectal cancer surgeries, slightly lower than the 74% reported by Mehta OH et al., but still alarmingly high. Therefore, enhanced temperature management is crucial for these patients. This study aims to investigate the application of the wireless temperature monitoring and intelligent insulation management system in laparoscopic radical resection of rectal cancer. We hope to explore its effectiveness in integrated intraoperative temperature monitoring and warming management, provide reference for developing personalized and scientific temperature management protocols, and support the construction of a perioperative temperature management database.

**II. Research Objectives**

1. To investigate the effectiveness of the wireless temperature monitoring and intelligent insulation management system in preventing intraoperative and postoperative hypothermia in patients undergoing laparoscopic radical resection of rectal cancer. This study aims to provide reference for developing personalized and scientific temperature management protocols for these patients and to support the construction of a perioperative temperature management database.

2. To explore the warming effects of different temperature management strategies in laparoscopic radical resection of rectal cancer. The goal is to identify a safe, applicable, efficient, and cost-effective temperature management model, thereby providing guidance for the selection of clinical temperature management protocols.

**III. Research Overview**

### 3.1 General Research Design and Plan

This study is a prospective, single-blind, randomized, controlled trial.

### 3.2 Study Population

#### 3.2.1 Inclusion Criteria

1. Preoperative pathological confirmation of rectal cancer with a planned laparoscopic radical resection.

2. Age ≥ 18 years.

3. American Society of Anesthesiologists (ASA) physical status classification of I to III.

4. Tympanic temperature in the morning of the surgery day is < 37.5°C or ≥ 36.0°C.

5. No ulcers, wounds, bleeding, or exudation in the axillary region.

6. Willingness to participate in the study with informed consent obtained.

#### 3.2.2 Exclusion Criteria

1. Patient refusal or non-cooperation.

2. Abnormal thermoregulation, such as malignant hyperthermia or neuroleptic malignant syndrome.

3. Presence of infectious fever within three days before surgery.

4. Confirmed hypothyroidism or hyperthyroidism.

5. Emergency surgery.

#### 3.2.3 Criteria for Withdrawal During the Study

1. Conversion from laparoscopy to open surgery.

2. Intraoperative occurrence of massive bleeding, shock, cardiac arrest, or respiratory arrest.

3. Postoperative bypass of the recovery room and direct transfer to the ward or ICU.

### 3.3 Sample Size and Grouping Methods

#### 3.3.1 Grouping Method

Patients meeting the inclusion and exclusion criteria will be consecutively enrolled and numbered (1-90) according to their order of enrollment. Using SPSS 25.0, 90 random numbers within the range of (0-1) will be generated and matched with patient numbers. Patients will be randomized into three groups, each consisting of 30 participants. The random allocation sequence will be placed in sequentially coded, sealed, and opaque envelopes. When researchers confirm the eligibility of a subject, the corresponding envelope will be opened, and the subject will be assigned to the designated group.

#### 3.3.2 Trial Groups

**1. Routine Group**: Conventional warming methods will be used, including maintaining the operating room temperature at 23-24°C and humidity at 40%-60%. Patients will be covered with cotton blankets over non-surgical areas before anesthesia and throughout the surgery. The temperature of intraoperative abdominal irrigation fluid will be maintained at 37°C to 38°C.

**2. Closed-Loop Thermoregulation Group**: In addition to conventional warming, this group will utilize a wireless temperature monitoring and intelligent warming management system. The patient warming system in the operating room bed will be preheated for 15 minutes before the patient enters the room, reaching a preset temperature of 38°C. Patients will lie on the preheated warming pad upon entering the operating room, and a warming blanket will be applied until it is removed before surgical skin disinfection. During surgery, the warming device's output power will be automatically adjusted based on real-time core temperature data from the wireless temperature sensors to meet individualized intraoperative temperature targets.

**3. Enhanced with Warming Blanket Group**: This group will receive forced air warming in addition to conventional methods. The warming device will be set to 38°C and activated upon patient entry into the operating room. The forced air warming blanket will be turned off at the end of the surgery.

**3.4 Study Procedures and Related Examinations**

#### 3.4.1 Screening Period

The day before surgery, the surgical schedule will be reviewed, and eligible patients will be visited preoperatively. The study's purpose, implementation process, and key points will be explained to the patients. Those who agree to participate will sign the informed consent form. On the morning of the surgery, patients will be re-evaluated to confirm their eligibility for enrollment.

#### 3.4.2 Enrollment and Treatment Period

1. Patients will be admitted to the preoperative waiting area 30 minutes before surgery. The temperature and humidity of the waiting area and operating room will be preset at 22-24°C and 40%-60%, respectively. All patients in the waiting area will be provided with cotton blankets for passive warming.

2. Upon entering the operating room, a wireless temperature sensor will be placed deep in the patient's axilla, close to the axillary artery, and secured with a dedicated adhesive patch. Patients and circulating nurses will be instructed to keep the patient's arm adducted for 8 minutes to ensure accurate initial temperature data. The sensor will remain in place until the patient leaves the post-anesthesia care unit (PACU), with temperature data continuously transmitted to a data receiver. Temperature management will be conducted according to the assigned protocol.

#### 3.4.3 Data Collection

**1. Demographic Data**: Basic patient information, including age, gender, height, and weight, will be collected preoperatively.

**2. Surgical Information**: Preoperative diagnosis, surgical procedure, anesthesia method, incision classification, anesthesia grading, intraoperative diagnosis, surgical name (as confirmed postoperatively), intraoperative fluid balance, and volume of intraoperative irrigation.

**3. Time Points**: Entry into the operating room, placement of the wireless temperature sensor, anesthesia induction, intubation, skin incision, wound closure, exit from the operating room, entry and exit from the PACU, anesthesia recovery time, extubation time, and duration of PACU stay.

### 3.5 Endpoint Measures

**1. Core Body Temperature**: Core body temperature will be continuously monitored using the wireless temperature monitoring system from the time the patient enters the operating room until leaving the PACU, with recordings taken every 15 minutes.

**2. the occurrence of hypothermia:** The number of patients who develop hypothermia during the period from entering the operating room to leaving the PACU.

**3. Duration of Hypothermia**: The total duration of hypothermia as a percentage of the total time from entering the operating room to leaving the PACU.

**4. Time to tracheal extubation**: Extubation time will be recorded from the end of skin suturing (when the wound dressing is applied and the patient is placed in a supine position) to the time of extubation.

**5. Length of stay in the PACU**: The duration of stay in the PACU will be calculated based on entry and exit times.

**6. Incidence of postoperative shivering**: The occurrence and severity of shivering will be assessed using Wrench's four-level grading scale: Grade 0—no visible muscle activity; Grade 1—piloerection, peripheral vasoconstriction, or both (other causes excluded); Grade 2—moderate shivering with visible muscle tremors; Grade 3—severe shivering with obvious full-body shaking.

**IV. Adverse Event Monitoring**

### 4.1 Definition of Adverse Events

#### 4.1.1 Definitions

**1. Adverse Event**: An unfavorable medical occurrence in a patient or clinical trial subject after receiving a medication or intervention. However, it does not necessarily have a causal relationship with the treatment.

**2. Serious Adverse Event**: An event occurring during a clinical trial that requires hospitalization, prolongs hospital stay, results in disability, affects work capacity, is life-threatening, or leads to death, or causes congenital anomalies.

#### 4.1.2 Severity Grading

**1. Mild**: The subject can tolerate it without affecting treatment. No special intervention is required, and it has no impact on the subject's recovery.

**2. Moderate**: The subject finds it difficult to tolerate, requiring special intervention, and it directly affects the subject's recovery.

**3. Severe**: Life-threatening to the subject, potentially fatal or disabling, and requires immediate emergency treatment.

### 4.2 Recording and Reporting of Adverse Events

Adverse events, including serious adverse events, may occur during the treatment process. If an adverse event occurs, it should be meticulously documented in the case report form, including the time of occurrence, clinical manifestations, management process, duration, outcome, and relationship to the intervention. For subjects with laboratory abnormalities, follow-up should continue until the results return to normal, reach pre-treatment levels, or are determined to be unrelated to the study intervention. In the event of a serious adverse event, a Serious Adverse Event Report must be completed and reported to the sponsor, ethics committee, CFDA (China Food and Drug Administration) Safety Supervision Department, and relevant health authorities within 24 hours.

### 4.3 Risk Prevention and Management

**1. Patient Protection**:

Ensure proper sterilization and disinfection of trial equipment.

Strictly adhere to the inclusion criteria of the study protocol to ensure the safety of subjects and prevent cross-infection.

1. **Research Equipment Management**:

Regular maintenance and inspection of equipment to ensure proper functioning.

Implement timely equipment upgrades and ensure backup devices are available for use in case of unexpected situations to prevent interruptions in the trial process and ensure the smooth implementation of the study.

**5.1 Sample Size Estimation**

The sample size was estimated using the formula for a parallel-group design: n=Δλand $\triangle=\frac{1}{\sigma^{2}}\sum_{i=1}^{k} \left(\text{u}_{\text{i}}-\text{u}_{0}\right)^{2}$
where:

n represents the sample size per group;

σ represents the standard deviation;

k represents the number of groups;

ui​ represents the mean of each group;

u0​ represents the overall mean of the group means.

With α=0.05, β=0.1, and k=3, the value of λ was obtained from the table as 12.66. Based on the primary outcome measure (core temperature) from the pilot study, the sample size was calculated. The pilot study results were: u1​=36.55, u2​=36.16, u3​=35.84, and σ=0.65. The overall mean u0​ was calculated as 36.18. Substituting these values into the formula, the sample size per group was estimated to be n=22. Considering potential dropouts and the need for effective data collection, the sample size for each group was set at 30 participants.

**5.2 Statistical Analysis of Research Data**

**1. Descriptive Statistics**: General demographic data of the three groups will be described using means and standard deviations, frequencies and percentages, and medians with interquartile ranges. For continuous data in the general demographic information, analysis of variance (ANOVA) will be used. For categorical data, chi-square tests and Kruskal-Wallis tests will be employed as appropriate.

**2. Inferential Statistics**: Comparison of core temperature at the same time point among the three groups will be analyzed using one-way ANOVA. For repeated measurements of core temperature over multiple time points, repeated-measures ANOVA will be used. The incidence and duration of hypothermia, as well as the incidence of postoperative shivering, will be compared among the three groups using chi-square tests. Postoperative extubation time and PACU (Post-Anesthesia Care Unit) stay duration will be compared among the three groups using ANOVA.

**VI. Ethical Considerations**

### 6.1 Ethical Review by the Ethics Committee

The study protocol, written informed consent form, and all materials directly related to participants must be submitted to the Ethics Committee for review. The study can only be initiated after obtaining written approval from the Ethics Committee. Researchers must submit an annual report to the Ethics Committee (if applicable). Upon termination or completion of the study, researchers must notify the Ethics Committee in writing. Any changes occurring during the study (e.g., revisions to the protocol or informed consent form) must be promptly reported to the Ethics Committee and cannot be implemented until approved, except for changes made to eliminate apparent and immediate risks to participants. In such cases, the Ethics Committee will be notified accordingly.

### 6.2 Informed Consent

#### 6.2.1 Procedure for Obtaining Informed Consent

Researchers must provide participants or their legal representatives with an easily understandable informed consent form, which has been approved by the Ethics Committee. Participants must be given ample time to consider their involvement in the study. Enrollment of participants will not proceed until a signed, written informed consent form is obtained. During the study, participants will be provided with any updated versions of the informed consent form and related information. The informed consent form should be retained as an essential document for the clinical trial.

**VII. Confidentiality Measures**

Results from this study may be published in medical journals. However, we will comply with legal requirements to protect patient information and ensure confidentiality. Personal information will not be disclosed unless required by law. When necessary, regulatory authorities, the hospital ethics committee, and relevant personnel may access patient data in accordance with established regulations.

**VIII. Anticipated Timeline and Completion Date**

June 2020 – October 2020: Formal experimental research will commence, following the study protocol with strict quality control to ensure the authenticity and reliability of the research.
