# Supplementary material for: Closed-loop temperature management with internet of things technology support in elderly laparoscopic rectal cancer surgery: A randomised controlled trial
Source: PLoS One. 2025 Nov 13;20(11):e0335993. doi: 10.1371/journal.pone.0335993 (PMC12614596; doi:10.1371/journal.pone.0335993)
Supplement: S3 File — (DOC) [file pone.0335993.s003.doc]

**研究方案**

**项目名称（中文）：无线体温监测与智能保温一体化管理模式在腹腔镜直肠癌根治术中的应用研究**

**项目名称（英文）：Effects of wireless temperature monitoring and intelligent insulation management to Patients undergonig Laparoscopic radical resection of rectal cancer.**

研究单位：西南医科大学附属医院

研究负责人：王雪

申办者：西南医科大学附属医院

1. **研究背景**

体温是重要的生命体征之一，保持体温恒定是保证机体新陈代谢和正常生命活动的必要条件，而体温异常则会引起代谢功能紊乱甚至危及生命。手术患者由于受手术室的特殊环境、手术部位暴露、手术操作、麻醉因素、术中输入大量常温或低温液体以及术野冲洗等因素易发生术中或术后低体温。但由于目前常规的体温监测方法存在局限性以及保温设备配置有限等原因，使患者术中体温监测落实率低、保温评估与实施缺乏循证而出现保温不足或过度保温的现象，以至给患者术后康复带来严重影响。目前基于互联网、AI技术将病员加温系统与无线体温监测系统联合应用形成的智能体温监测与保温闭环管理系统已经能实现体温监测与保温的一体化管理；其中无线体温传感器通过蓝牙转换器传输体温数据至Warm6200的制器，控制器通过患者的实时体温数据管理患者体温。这对临床体温管理理念、方法、管理结果将会带来革命性的改进，但其临床应用效果如何，且如何用好设备、发挥设备效能、服务于患者，提升科学化管理水平，均是围手术团队期待与实施探索的热点和研究课题。

另外本研究团队前期的横断面调查显示，其中结直肠癌术中低体温的发生率为66.3%，略低于Mehta OH等人的调查结果（74%），但其发生率依然很高。因而，更需要加强对该类患者的体温管理。综上，本研究拟定为：无线体温监测与智能保温一体化管理模式在腹腔镜直肠癌根治术中的应用研究。以期探索该应用模式对腹腔镜直肠癌患者术中体温监测与保温一体化管理效果，为制定腹腔镜直肠癌患者术中个性化科、学化体温管理方案提高参考依据，为下一步围手术期体温管理数据库的构建提供数据支持。

**二、研究目的**

1. 探索无线体温监测与智能保温一体化系统管理模式对的腔镜直肠癌患 者术中和术后复苏期低体温预防的效果，为制定腹腔镜直肠癌患者术中个性化、科学化体温管理方案提高参考依据，为下一步围手术期体温管理数据库的构建提供数据支持。
2. 探讨不同体温保温方案在腹腔镜直肠癌根治术中的保温效果，探寻安全、适用、高效、经济的的体温管理模式，为临床体温管理实施方案的选择提供参考。

**三、研究概况**

3.1整体的研究设计和计划

本研究为一项前瞻性、单盲、随机、对照研究。

3.2 研究人群

3.2.1入选标准

1. 术前病理证实为直肠癌，术前评估拟行腹腔镜直肠癌根治手术；
2. 年龄≥18岁；
3. 美国麻醉医师协会（American Society of Anesthesiologists ,ASA）Ⅰ ～ Ⅲ 级；
4. 术日晨鼓膜温度低于37.5℃或者≥36.0℃；
5. 腋下无溃疡、伤口、涉血、渗液者；
6. 自愿参与本研究，并签署知情同意书。

3.2.2排除标准

1. 患者拒绝或不合作；
2. 体温调节异常，如恶性高热、抗精神病药恶性综合征；
3. 术前3天有感染性发热的患者；
4. 明确证据确诊的甲减和甲亢；
5. 急诊手术。

3.2.3中途退出标准

1. 术中腔镜中转开腹患者；
2. 术中出现大出血、休克、呼吸心跳骤停等；
3. 术后未进入复苏室，直接回到病房或ICU的患者。

3.3病例数及分组方法

3.3.1分组方法

将符合纳入、排除标准的患者连续入组，即根据患者纳入顺序对患者进行编号（1-90），利用spss25.0产生（0-1)范围内的90个随机数并与患者编号一一对应，并随机化分为三组，每组30例。产生的随机分配序列放入按顺序编码，密封，不透光的信封中，当研究人员确定受试对象的合格性后，按顺序拆开信封并将受试对象分配入相应的试验组。

3.3.2试验分组

1. 常规保温组

采用常规保温方法：室温恒定为 23-24℃、湿度为 40%～60%，麻醉前和手术中患者手术区以外身体部位均用棉被覆盖保温至手术结束，术中腹腔冲洗液加温温度为37℃—38℃。

1. 无线体温监测与一体化管理模式保温组

在常规保温基础上，采用无线体温监测与智能保温一体化管理系统进行保温，患者进入手术间前将手术床上的病员加温系统中的盖毯和垫毯预热15min，使其加热温度达到预设保温值38℃，患者便进入手术间躺在已预热手术床加温垫上，同时加盖加温毯至手术皮肤消毒前退去加温盖毯。手术中，根据无线体温监测传感器对患者核心体温的持续动态的实时监测数据反馈，自动调节加温设备输出功率，满足患者术中个性化体温设定目标需求，进行术中保温。

1. 充气加温组

在常规保温的基础上，采用充气加温装置保温，患者进入后便给予加温，充气加温装置的档位调至38℃，待手术结束后关闭充气加温毯。

3.4研究步骤及相关检

3.4.1筛选期

术前一天查看手术安排程，分别记录符合研究对象的患者进行术前访视，向患者说明研究的目的，研究实施配合过程与要点，同意参与的患者填写知情同意书；术晨再次对患者进行评估，最终确定入组患者。

3.4.2入选治疗期

1. 患者于术前30 min接入手术室等候区，将等候区与手术室温度预先设定为 22-24℃，湿度为40%～60%，在等候区所有患者均给予棉被进行被动保暖。
2. 患者进入手术间后，将无线体温传感器紧贴于患者腋下深部靠近腋动脉处，通过专用敷贴固定传感器，同时告知患者和巡回护士，保持患者手臂夹闭8分钟，以促进传感器的对患者初始体温的真实数据，并通过数据盒连接传感器，将体温监测的数据实时传输到数据盒，传感器直至患者出麻醉复苏室后取出；并根据相应的体温管理方式进行体温管理。

3.4.3相关资料收集

1. 一般资料：术前收集包括年龄、性别、身高、体重等患者基本资料；
2. 手术相关信息 : 术前诊断、手术方式、麻醉方式，切口分级，麻醉分级，术中诊断，手术名称（术后确定的名称），患者术中液体出入量、术中冲洗量；
3. 时间节点：患者入室时间、无线体温传感器安置时间、麻醉时间、插管时间、切皮时间、切口缝合时间、出手术室时间、出入复苏室时间、麻醉恢复时间，拔管时间及复苏室停留时间停留时间。

3.5 终点指标

1. 患者的核心体温：从患者进入手术间到离开PACU之间，采用无线体温监测系统实时采集患者核心体温，且每15min记录一次患者核心体温。
2. 低体温发生率：从患者进入手术间到离开PACU之间低体温的发生人数；
3. 低体温发生时长占比：从患者进入手术间到离开PACU之间低体温发生的总时长占比；
4. 苏醒拔管时间：患者进行皮下缝合结束，待切口敷贴粘贴完毕，将患者恢复仰卧位时作为手术结束时间点，记录患者拔管时间，计算患者的苏醒拔管时间（拔管时间－点－手术结束时点）；
5. 麻醉恢复室滞留时间：记录进出麻醉复苏室的时间，计算患者术后麻醉恢复室停留时间。
6. 寒战：寒颤的发生情况及寒战分级，寒战的评估采用 Wrench^[39]^的四级评估量表进行未见明显肌肉活动。0级一无寒战发生；1级一除了立毛，外周血管收缩，或两者同时存在(已排除其他原因）；2级一中度寒战肌肉可见明显的颤抖；3级—重度寒战，整个身体明显抖动。

**四、不良事件观察**

4.1 不良事件的定义

4.1.1定义

不良事件：病人或临床试验受试者接受一种药品后出现的不良医学事件，但并不一定与治疗有因果关系。

严重不良事件：临床试验过程中发生需住院治疗、延长住院时间、伤残、影响工作能力、危及生命或死亡、导致先天畸形等事件。

4.1.2程度

轻度：受试者可忍受，不影响治疗，不需要特别处理，对受试者康复无影响。

中度：受试者难以忍受、需要特殊处理，对受试者康复有直接影响。

重度：危及受试者生命，致死或致残，需立即做紧急处理。

4.2不良事件的记录及报告途径

受试者治疗过程中可能发生临床不良事件，一旦发生不良事件（包括重要不良事件），应在病例报告表上详细记录不良事件的发生时间、临床表现、处理经过和持续时间、转归以及与药物的关系；出现实验室检查异常者，须随访患者至检查结果恢复正常，或至用药前水平，或确定与试验药物无关。发生严重不良事件应填写严重不良事件表，并在24小时内报告申办者、伦理委员会、CFDA安监司和卫生行政部门。

4.3 风险的防范和处理

1. 患者保护：做好试验设备的消毒灭菌工作，严格按照试验方案纳入研究对象，保证研究对象的安全，避免交叉感染；
2. 研究设备：定时维护、检修设备，做好设备的更新换代工作，同时做好备用设备工作，意外情况发生随时使用备用设备，确保试验过程不被打断，保证研究的顺利实施。

**五、统计分析**

5.1样本含量估计

采用多组平行对照的设计计算公式 ：**n=λ/Δ**


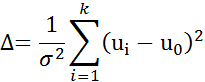


公式中：n 代表每组样本含量，σ代表标准差，k 代表组数，ui代表各组平均数，uo代表各组平均数的平均数。取a=0.05，b=0.1，k=3，查表可得 λ=12.66，根根据预实验中的主要结局指标核心温度计算样本量。经预实验u_1_=36.55，u_2_=36.16，u_3_=35.84，σ=0.65，可算u_0_=36.18，带入公式可得n=22，考虑到样本脱落和数据收集的有效性，每组取 30 人。

5.2研究数据的统计与分析

1. 描述性统计分析：三组患者一般资料用均数与标准差，频数与百分比，中位数与四分位数距描述；一般资料中的计量资料和计数资料分别采用方差分析、卡方检验和 Kruskal-Walli 检验。
2. 统计推断：三组患者同一时间点的核心温度比较采用单因素方差分析，多个时间点核心温度、采用重复测量方差分析；三组患者低体温发生率及发生时长占比和术后寒战发生率采用卡方检验，三组术后苏醒拔管时间、复苏室停留时间、采用方差分析。

**六、研究相关伦理学**

6.1伦理委员会审核

本方案和书面知情同意书及与受试者直接相关的资料必须提交伦理委员会，获得伦理委员会书面批准后方可正式开展研究。研究者必须至少每年（如果适用）向伦理委员会提交研究年度报告。在研究中止和/或完成时，研究者必须书面通知伦理委员会；研究者必须及时向伦理委员会报告所有研究工作中发生的变化（如方案和/或知情同意数的修订），并且在未获得伦理委员会批准之前不得执行这些变动，除非是为了消除对受试者明显且直接的风险而做出的变更。在发生这类情况时，将通知伦理委员会。

6.2知情同意

6.2.1获得知情同意的程序

研究者必须向受试者或其法定代理人提供易于理解的并且经伦理委员会批准的知情同意书，并给与受试者或其法定代理人充分的时间考虑本项研究，在从受试者获得签署的书面知情同意书之前，受试者不得入组。 在受试者参与期间，将向受试者提供所有更新版本的知情同意书以及书面信息。知情同意书应作为临床试验的重要文档保留备查。

**七、保密措施**

通过本项目研究的结果可能会在医学杂志上发表，但是我们会按照法律的要求为患者的信息保密，除非应相关法律要求，患者的个人信息不会被泄露。必要时，政府管理部门和医院伦理委员会及其有关人员可以按规定查阅患者的资料。

**八、研究项目的预期进度和完成日期:** 2020.06--2020.10：开始正式实验研究，按照实验方案进行，并注意过程中质量控制，保证研究的真实性和可靠性。

1. **参考文献**

[1]National Institute For Health And Clinical Execllence. Hypothermia: prevention and management in adults having surgery[CG65] [2019-2-11]. https://www.nice.org.uk/guidance/cg65.

[2]Vural F，çelik B，Deveci Z，et al. Investigation of inadvertent hypothermia incidence and risk factors[J]. Turkish Journal of Surgery，2018，34(4): 300-305.

[3]Yi J，Lei Y，Xu S，et al. Intraoperative hypothermia and its clinical outcomes in patients undergoing general anesthesia: National study in China[J]. PLOS ONE，2017，12(6): e177221.

[4]Yi J，Xiang Z，Deng X，et al. Incidence of Inadvertent Intraoperative Hypothermia and Its Risk Factors in Patients Undergoing General Anesthesia in Beijing: A Prospective Regional Survey[J]. PLOS ONE，2015， 10(9): e136136.

[5]徐彦，陈茜，陆建平，等. 术后苏醒室低体温发生率及危险因素[J]. 复旦学报(医学版)， 2016， 43(03): 302-307.

6]Hooper V D，Chard R，Clifford T，et al. ASPAN's evidence-based clinical practice guideline for the promotion of perioperative normothermia: second edition[J]. J Perianesth Nurs，2010，25(6): 346-365.

[7]Bindu B，Bindra A，Rath G. Temperature management under general anesthesia: Compulsion or option[J]. J Anaesthesiol Clin Pharmacol，2017，33(3): 306-316.

[8]冯腾尘，崔晓光. 围术期低体温防治研究进展[J]. 中华实用诊断与治疗杂志, 2016，30(03): 218-221.

[9]薛莹. 围手术期低体温防治的研究进展[J]. 护士进修杂志，2015，30(21): 1938-1941.
